# An Anatomorphometric Study of Occipital Spurs and Their Association With Dental Occlusion

**DOI:** 10.7759/cureus.51827

**Published:** 2024-01-07

**Authors:** Fatma Çağlayan, Berfin Polat, Hatice L Tugluoglu Dalci, Esra Oncu, Nida Kuzey, Hatice Guller

**Affiliations:** 1 Oral and Maxillofacial Radiology, Faculty of Dentistry, Ataturk University, Erzurum, TUR; 2 Pediatric Dentistry, Faculty of Dentistry, Izmir Katip Celebi University, Izmir, TUR

**Keywords:** diagnostic imaging, dental occlusion, spur, osteophyte, occipital bone

## Abstract

Background and objective

The occipital spur (OS) can be described as an abnormal elongation of the external occipital protuberance (EOP); its etiology is multifactorial and may involve biomechanical, immunological, and/or genetic factors. This study aimed to determine the frequency and characteristics of elongated EOP or OS as well as the relationship of OS with occlusion in a group of Turkish dental patients.

Materials and methods

Lateral cephalometric radiographs of 1925 patients taken in 2022 were retrospectively analyzed. The frequency, types, and dimensions of OS were determined based on gender and age groups. Molar occlusion and incisal bite were also evaluated.

Results

Of the 1925 patients, 679 were males and 1246 were females. The mean age of the cohort was 18.17 ±5.03 years (range: 4-61). OS was detected in 483 (25.1%) patients and was more common in males (p<0.001); 133 (27.5%) of the OSs were flat, 247 (51.1%) crest, and 103 (21.3%) spin type. The incidence of OS increased depending on age groups (p<0.001). There was no statistically significant association between OS presence and molar occlusion (p>0.05). However, a statistically significant association was observed between anterior incisal bite (p=0.001) and OS presence. There was a statistically significant difference in terms of OS sizes in males and females; the sizes of OS were larger in males than in females (length: p<0.05, base and thickness: p<0.001).

Conclusions

The frequency of OS was quite high in our cohort; it was more common and of larger size in males and older age groups. The most common type was the crest type. While there was no statistically significant association between OS frequency and molar occlusion, there was a significant relationship with incisal bite. The frequency of OS was highest in people with anterior crossbite.

## Introduction

The occipital bone is a circular membranous flat bone found in the posterior and inferior part of the cranium. The basilar and lateral parts of the occipital bone form the boundaries of the foramen magnum. There is a bulge called external occipital protuberance (EOP) on the middle of the outer surface of the occipital bone. Ligamentum nuchae, which is an important, triangular, yellow, elastic structure that is the continuation of the ligamentum supraspinale and prevents the head from falling down, is attached near to the EOP [1]. The trapezius muscle also arises from the EOP [2,3].

Bone protrusions formed as a result of this inflammation are called spurs. The pathogenesis of spurs is multifactorial and may involve biomechanical, immunological, and/or genetic factors. Disturbances in calcium metabolism may also play a role. They can also be thought of as a functional adaptation resulting from mechanical stresses [4,5]. The occipital spur (OS) refers to the abnormal elongation of the EOP [3] and is also termed occipital knob, occipital bun, chignon or inion hook, and elongated or enlarged EOP [6]. Elongated EOP or OS is quite common and is classified into three types: Type I: smooth, Type 2: crest, and Type 3: spine [6,7].

Shahar et al. [4] have suggested that mechanical load has an important role in OS development regardless of whether inflammatory or genetic factors are involved. According to one hypothesis, this type of bony prominence occurs more commonly in people who frequently engage in vertical movements such as playing basketball/volleyball and in climbing athletes, due to the overuse and overstretching of the trapezius muscle [2,3]. It has been reported that it is associated with certain issues such as occipital headache, neck pain, and limitation of movements in the shoulders and arms [3].

In recent years, EOP elongation or OS has been encountered frequently in radiological images. In this study, we aimed to determine the frequency and characteristics of OS on lateral cephalometric films, as well as the relationship of OS with dental occlusion in a group of Turkish dental patients. Although a few studies investigating the frequency of OS are available in the literature, there are no studies evaluating OS together with occlusion. A relationship between cranial shape and occlusion has been observed in the literature [8].

## Materials and methods

Study design

This cross-sectional retrospective study was conducted in the Department of Oral and Maxillofacial Radiology in 2022. Ethical approval was obtained from the Ethics Committee of the Faculty of Dentistry (decision number: 2022/88). Six researchers conducted the study and three researchers performed the radiographic evaluations. The researcher who spearheaded the study, a physician with 20 years of experience in maxillofacial radiology, trained the research team on how to make assessments and measurements. The first 30 radiographs were evaluated together under the leadership of the head, and the researchers calibrated each other. In addition, inter-observer agreement was tested. A total of 1925 cephalometric radiographs taken in 2022 in the Department of Oral and Maxillofacial Radiology were evaluated retrospectively. We excluded radiograms with poor diagnostic quality, radiograms of patients receiving orthodontic treatment, and radiograms with advanced craniofacial disorders.

X-ray parameters

The study included cephalometric radiographs taken in our department in 2022 with the same device and involving the same parameters. Radiographs were taken with the Planmeca Promax 2D S2 (Planmeca Oy; Helsinki, Finland) device by employing the following parameters; 66 kVp, 10 mA, and 10.5 s. The patients were positioned with the sagittal line perpendicular to the ground and the canthomeatal line parallel to the ground, with the ear sticks fully inserted during the scanning. The beam tube remained on the patient's left side with the sensor on the right.

Evaluation of occipital spurs

**Figure 1:**
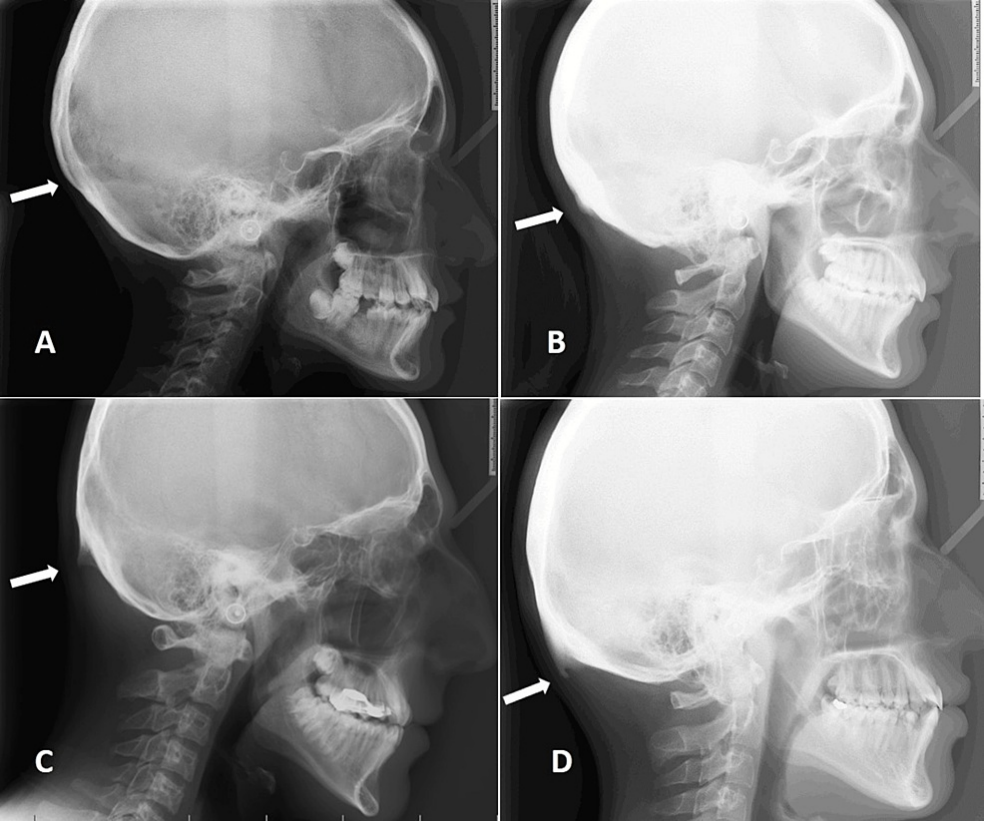
Presence and types of occipital spurs A: none; B: Type 1 (flat); C: Type 2 (crest); D: Type 3 (spin)

**Figure 2:**
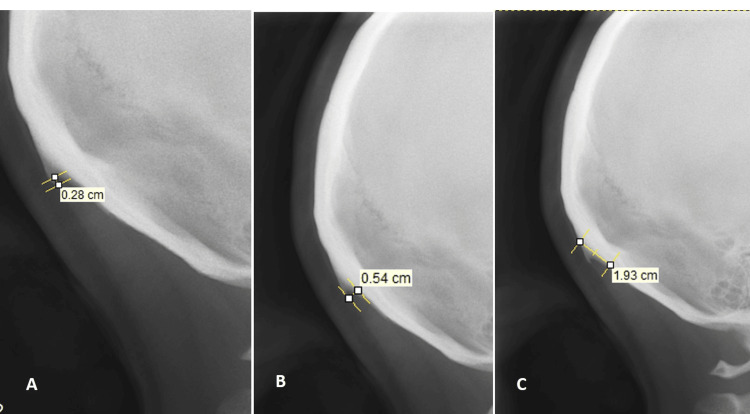
Measurements of the occipital spur A: length; B: base; C: thickness

The archive was scanned using the Turcasoft software (Turcasoft Dent, Samsun, Turkey). Firstly, the presence of OS was evaluated. If OS was present, its type had been detected. OSs were classified as follows: Type 1 (flat), Type 2 (crest), and Type 3 (spine) as previously reported in the literature (Figure 1) [6,7]. Then, measurement procedures were initiated in OS-positive patients. As part of the measurements, the base and thickness of the OS and the length in Type 3 were also measured (Figure 2). Measurements were made directly with the measuring stick in the automation program used and the results were recorded in mm.

Evaluation of occlusion

While evaluating the occlusion, molar and incisal relations were considered. The Angle's classification was used for the evaluation of molar occlusion. This classification is based on the anterior-posterior relationship of the upper and lower first molars. Accordingly, anomalies are classified into three categories: Class I, Class II, and Class III. Apart from utilizing Angle's classification, the relationship of the lower and upper incisors was also evaluated separately. Accordingly, variants such as normal, open bite, deep bite, crossbite, teeth to teeth, and increased over-jet were documented.

Statistical analysis

IBM SPSS Statistics version 20 (IBM Corp., Armonk, NY) was used for statistical analysis. The chi-square test was used to determine the distribution and for the statistical analysis of OS frequency and types depending on gender, age group, occlusion, and incisal bite. For all analyses, expected counts of cells below 5 and distributions were deemed suitable for the test. The independent sample t-test was used to compare OS sizes between males and females. Levene’s test was employed to evaluate the homogeneity of data. The one-way ANOVA test was used to compare OS sizes across age groups. A p-value <0.05 was considered statistically significant.

## Results

Cephalometric radiographs of 1925 patients - 679 males and 1246 females - were included in the study. The mean age of the cohort was 18.17 ± 5.03 years (range: 4-61 years). OS was detected in 483 (25.1%) patients. Of these, 133 (27.5%) were Type 1 (flat), 247 (51.1%) were Type 2 (crest), and 103 (21.3%) were Type 3 (spin). The distribution of OS frequency and types based on gender, age, occlusion, and incisal bite is shown in Table 1. The incidence of OS was higher in males than females (p˂0.001); while OS was observed in 18.4% of females, it was observed in 37.1% of males. The most common type in both females and males was the crest type (Type 2).

**Table 1:** Distribution of OS frequency and types according to gender, age group, occlusion, and incisal bite ^a^Chi-square test; *p=0.001; **p˂0.001 OS: occipital spur

Variables	Occipital spur				Total, n (%)	P-value^a^
	None, n (%)	Type 1, n (%)	Type 2, n (%)	Type 3, n (%)		
Gender						
Male	425 (62.9)	54 (8.0)	149 (21.9)	51 (7.5)	679 (100)	0.000**
Female	1017 (81.6)	79 (6.3)	98 (7.9)	52 (4.2)	1246 (100)	
Total	1442 (74.9)	133 (6.9)	247 (12.8)	103 (5.4)	1925 (100)	
Age group, years						
14-Apr	378 (93.1)	14 (3.4)	10 (2.5)	4 (1.0)	406 (100)	0.000**
15-24	971 (71.1)	101 (7.4)	206 (15.1)	88 (6.4)	1366 (100)	
25-34	84 (62.7)	14 (10.4)	27 (20.1)	9 (6.7)	134 (100)	
˃35	9 (47.4)	4 (21.1)	4 (21.1)	2 (10.5)	19 (100)	
Total	1442 (74.9)	133 (6.9)	247 (12.8)	103 (5.4)	1925 (100)	
Occlusion						
Class I	454 (76.6)	44 (7.4)	67 (11.3)	28 (4.7)	593 (100)	P>0.05
Class II	576 (75.0)	54 (7.0)	106 (13.8)	32 (4.2)	768 (100)	
Class III	412 (73.0)	35 (6.2)	74 (13.1)	43 (7.6)	564 (100)	
Total	1442 (74.9)	133 (6.9)	247 (12.8)	103 (5.4)	1925 (100)	
Incisal bite						
Normal	330 (72.4)	34 (7.5)	65 (14.3)	27 (5.9)	456 (100)	0.001*
Open bite	128 (71.5)	9 (5.0)	29 (16.2)	13 (7.3)	199 (100)	
Deep bite	242 (78.1)	31 (10.0)	23 (7.4)	14 (4.5)	310 (100)	
Crossbite	178 (70.1)	13 (5.1)	39 (15.4)	24 (9.4)	254 (100)	
Teeth to teeth	157 (77.3)	15 (7.4)	21 (10.3)	10 (4.9)	203 (100)	
Overjet	407 (77.8)	31 (5.9)	70 (13.4)	15 (2.9)	523 (100)	
Total	1442 (74.9)	133 (6.9)	247 (12.8)	103 (5.4)	1925 (100)	

When the distribution of OS frequency according to age groups was examined, statistically significant differences were observed (p<0.001). The incidence of OS increased depending on age groups. For example, while OS was observed in 6.9% of patients aged 4-14 years, it was observed in 52.6% of patients over 35 years. There was no statistically significant association between OS and molar occlusion (p>0.05). However, there was a statistically significant correlation between anterior incisal bite and the frequency of OS (p=0.001). Accordingly, the incidence of OS was highest in patients with anterior crossbite. Table 1 also shows the incidence of OS depending on the incisal bite shapes. The distribution of OS dimensions by gender is shown in Table 2. There was a statistically significant difference in OS sizes between males and females: the mean sizes of OSs were larger in males than females (p<0.05 for length, p<0.001 for base and thickness).

**Table 2:** Comparison of OS sizes depending on gender ^a^Independent sample t-test; *p<0.05; **p<0.001 OS: occipital spur; SD: standard deviation

		Number of OS	Mean ±SD, mm	t	P-value^a^
Length (Type 3)	Female	51	8.23 ±5.56	-2.526	0.014*
	Male	52	11.82 ±7.11		
Base (Types 1, 2, 3)	Female	229	11.44 ±6.84	-3.778	0.000**
	Male	254	14.44 ±7.69		
Thickness (Types 1, 2, 3)	Female	229	6.64 ±4.57	-4.775	0.000**
	Male	254	9.78 ±7.22		

The average OS size measurements in the entire study group were as follows - length: 10.05 ±6.61 mm; base: 12.91 ±7.41 mm; and thickness: 8.18 ±6.21 mm. The distribution of OS dimensions based on age groups is shown in Table 3. While there was no significant association of OS base width and length with age groups (p>0.05), a significant relationship was observed between OS thickness and age groups: OS thickness was highest in the age group of 15-24 years and lowest in the age group of 4-14 (p<0.05).

**Table 3:** Comparison of OS size based on age groups ^a^One-way ANOVA test; *p<0.05 OS: occipital spur; SD: standard deviation

		Number of OS	Mean ±SD, mm	P-value^a^	
					Length (Type 3)	4-14 years	4	5.43 ±6.49	0.088	
15-24 years	88	9.82 ±6.64							
25-34 years	9	14.79 ±4.57							
Over 35 years	2	5.85 ±5.02							
Base (Types 1, 2, 3)	4-14 years	28	10.13 ±6.84	0.252	
	15-24 years	395	12.00 ±7.42		
	25-34 years	50	14.06 ±7.93		
	Over 35 years	10	12.31 ±4.85		
Thickness (Types 1, 2, 3)	4-14 years	28	5.20 ±3.68	0.021*	
	15-24 years	395	8.68 ±6.29		
	25-34 years	50	6.49 ±4.91		
	Over 35 years	10	7.62 ±10.68		

## Discussion

An increase in the incidence of prolonged EOP or OS in young people due to the increase in the use of electronic devices and associated postural disorders has been recently observed. Moreover, researchers have carried out several studies on this subject. Jacques et al. [9] investigated the frequency of EOP elongation in young people due to the increase in smartphone use and postural disorders using CT scans in 2011 and 2019; they could not find a difference between the prolonged EOP between 2011 and 2019 (44.9% vs. 44.2%). The researchers also measured the EOP volumes and reported that they could not find any difference in values between the relevant years. In the same study, two XVIth-century young Egyptian mummy skulls were also scanned with CT, and EOP elongation was detected. The authors concluded that there was no increase in OS frequency despite the increase in smartphone usage over the years. Porrino et al. [10] also studied cervical spine radiographs taken in 2007 and 2017 to determine the effect of smartphone use on OS formation, and they found no relationship between the increase in smartphone usage and OS formation. In fact, they even found the frequency of OS to be higher in 2007 in total.

Since cephalometric films are two-dimensional, the length and thickness of the OS can be measured on these films while its width cannot be measured. OS size measurements in the present study were as follows - length: 10.05 ±6.61 mm; base: 12.91 ±7.41 mm; and thickness: 8.18 ±6.21 mm; our findings including OS dimensions generally align with other similar studies in the literature. The frequency of OS was found to be higher in radiological studies than in cadaver studies [11]. Srivastava et al. [3] found the frequency of OS to be 10% on 30 cadaveric skulls in 2018. They also measured the dimensions of the OS and found the mean width of the OS to be 13.40 mm, mean length to be 13.45 mm, and mean thickness to be 2.43 mm. In 2012, Singh [2], in his study of skulls with 40 EOPs, found an average EOP width of 6 mm, length of 8 mm, and thickness of 1.5 mm. Shahar and Sayers [5] found the prevalence of OS among young adults to be 40%. They also noted that 10% of them were more than 20 mm in length.

In a study conducted in our country in 2022, Gunacar et al. [7] found OS in 52% of people aged over 18 years and 47.5% of people aged under 18 years. In addition, they measured the mean OS length as 7.6 mm in women and 7.4 mm in men. They concluded that there was no significant difference in the frequency, type, and size of OS based on age groups and gender. However, we found the frequency of OS to be 25.1% and OS sizes were higher in males in the present study. The greater OS size in males can be attributed to sexual dimorphism. In addition, we found more OS tightness in older age groups in the present study. The reason for this may be that the effects of postural disorder, overuse, and stretching increase exponentially over time. Normally, spurs are rare in young people since it is thought that these skeletal changes occur gradually over time [4,12]. However, OS appears to be more common in young people [4,5]. This suggests a multifactorial etiology concerning OS formation. Posture disorders, which are common in today's youth, maybe the reason for this. One limitation of this study pertains to this aspect; since the study was conducted retrospectively on radiographs, we had no information about the habits, clinical conditions, and posture disorders of the patients. Hence, further studies are needed to investigate whether OS is related to these aspects as well as its symptoms.

OSs are usually asymptomatic but may occasionally cause symptoms such as occipital headache or infection in that area. Symptoms usually appear in late adolescence. This may be due to sub-periosteal stretching and tenderness that occurs during the growth of the protuberance. Headaches in the occipital and parietal regions can have many causes. Singh [2] stated that one of the reasons for these pains may be OSs. These protrusions can cause inflammation and spasms in the trapezius muscle and the occipital nerve may be irritated as well. As it is known anatomically, the third occipital nerve passes 3 mm lateral to the EOP [13,14].

Shahar et al. [4] performed hematological, genetic, and MRI examinations in people with OS longer than 30 mm, and they reported that there was no infection around OSs on MRI. They also did not find any relationship with genetic and infectious factors either. Satyarthee [13] presented a case of hornlike OS causing skin inflammation and occipital pain in a young patient. Such OSs may be vulnerable to trauma as well as these symptoms. Sattur et al. [15] reported an EOP fracture detected by CT. OS or prolonged EOP can also cause aesthetic complaints, especially in persons with baldness. OSs can be kept under control if they are asymptomatic. If they cause symptoms such as stinging or occipital headache, conservative approaches such as the use of soft pillows or analgesics may be considered. However, if the symptoms are persistent, they need to be surgically corrected [6]. Marshal et al. [16] reported three cases involving surgical treatment of symptomatic spine-type EOP.

Rauten et al. [8] observed that there was an association between dental-maxillary anomalies and cranial shapes. There is no other study similar to this study in the literature as no other studies have evaluated occlusion together with OS. Varghese et al. [6] reported a spine-type OS causing mild symptoms in a female patient with a Class III malocclusion. In the present study, there was no statistically significant difference between OS frequency and molar occlusion. However, there was a statistically significant correlation with anterior incisal bite. The Angle's classification, which evaluates molar occlusion, shows the relationship of the jaws in the sagittal direction. However, in incisal relations, the vertical position of the jaws is also taken into account. In the present study, while the frequency of OS was numerically the highest in individuals with Class III malocclusion (although not statistically significant), it was statistically highest in individuals with crossbite, which led us to think that positioning the mandible more anteriorly may change the head posture of the person and cause EOP elongation. Of course, to evaluate this situation more clearly, further studies evaluating face or head types, daily activities, and postures of people are required. However, this study may serve as a pioneer for such studies.

Forensic studies have reported that EOP is a landmark that can be used in detecting gender. According to Zhang and Schepartz [17], EOP is more pronounced in European men. OS dimensions were higher in males in the present study as well. Gülekon and Turgut [11], in their study on both skulls and radiological images in 2003, stated that the most common type of EOP in women was Type 1 (flat) while it was Type 3 (spin) in men, and the frequency of Type 2 was similar in both genders. Our study also found that the frequency of OS was higher in males; while OS was observed in 18.4% of women, it was observed in 37.1% of men. The most common type in both genders was the crest type (Type 2).

This study also has some limitations. Since the radiograms were evaluated retrospectively, data on the clinical status and lifestyle of the patients could not be obtained. Hence, we recommend further studies that involve long-term follow-up of OS. However, we believe that the relatively large sample size of this study is one of its strengths.

## Conclusions

Although the study was conducted in a particular patient group, the findings show that the frequency of OS is quite high. It was more common and of larger size in males and older age groups. The most common type was the crest type. While there was no statistically significant association between OS frequency and molar occlusion, there was a significant relationship with incisal bite. The frequency of OS was highest in people with anterior crossbite. Dentists and maxillofacial radiologists should be aware of OS and its symptoms, as well as the importance of correct head posture and reducing tension in the neck. In addition to correcting the occlusion and the relationship between the jaws, they can refer the patients to a relevant specialist for correcting the posture. We believe that our findings will also be useful for anatomists, anthropologists, archaeologists, forensic experts, neurologists, and maxillofacial surgeons.
